# Spontaneous formation of spiral-like patterns with distinct periodic physical properties by confined electrodeposition of Co-In disks

**DOI:** 10.1038/srep30398

**Published:** 2016-07-27

**Authors:** Irati Golvano-Escobal, Juan Carlos Gonzalez-Rosillo, Neus Domingo, Xavi Illa, José Francisco López-Barberá, Jordina Fornell, Pau Solsona, Lucia Aballe, Michael Foerster, Santiago Suriñach, Maria Dolors Baró, Teresa Puig, Salvador Pané, Josep Nogués, Eva Pellicer, Jordi Sort

**Affiliations:** 1 Departament de Física, Universitat Autònoma de Barcelona, E-08193 Bellaterra, Spain; 2 Institut de Ciència de Materials de Barcelona (ICMAB-CSIC), Campus UAB, E-08193 Bellaterra, Spain; 3 Catalan Institute of Nanoscience and Nanotechnology (ICN2), CSIC and The Barcelona Institute of Science and Technology, Campus UAB, Bellaterra, E-08193 Barcelona, Spain; 4 Institute of Microelectronics of Barcelona (IMB-CNM), Campus UAB, E-08193 Bellaterra, Spain; 5 Biomedical Research Networking Center in Bioengineering, Biomaterials and Nanomedicine (CIBER-BBN), E-08193, Bellaterra, Spain; 6 Alba Synchrotron Light Facility, CELLS, E-08280 Bellaterra, Spain; 7 Institute of Robotics and Intelligent Systems (IRIS), ETH Zürich, CH-8092 Zürich, Switzerland; 8 ICREA, Pg. Lluís Companys 23, 08010 Barcelona, Spain

## Abstract

Spatio-temporal patterns are ubiquitous in different areas of materials science and biological systems. However, typically the motifs in these types of systems present a random distribution with many possible different structures. Herein, we demonstrate that controlled spatio-temporal patterns, with reproducible spiral-like shapes, can be obtained by electrodeposition of Co-In alloys inside a confined circular geometry (i.e., in disks that are commensurate with the typical size of the spatio-temporal features). These patterns are mainly of compositional nature, i.e., with virtually no topographic features. Interestingly, the local changes in composition lead to a periodic modulation of the physical (electric, magnetic and mechanical) properties. Namely, the Co-rich areas show higher saturation magnetization and electrical conductivity and are mechanically harder than the In-rich ones. Thus, this work reveals that confined electrodeposition of this binary system constitutes an effective procedure to attain template-free magnetic, electric and mechanical surface patterning with specific and reproducible shapes.

The continuous progress in diverse areas of micro/nanotechnology relies on the ability to implement novel methods to create pre-defined patterned geometries at the surface of materials, with an ever-increasing resolution, using cost-effective, industrially-scalable methods. For this reason, lithographic techniques remain as core tools in the fields of electronics, photonics, microfluidics, or magnetic recording, among others^1^,^2^,^3^,^4^,^5^,^6^,^7^.

Surface patterning processes can be divided into two main categories^7^: (1) template/mask-assisted patterning and (2) mask-less pattern formation. Template-assisted patterning usually requires multiple processing steps: (i) creation of the templates or masks using, for example, electron beam or photolithography, (ii) thin film deposition through sputtering, electrodeposition or other methods, and (iii) physical or chemical etching of the mask to release or reveal the synthesized micro- or nanostructures. This multi-step procedure is often costly and complex as it requires the use of clean room facilities and sophisticated experimental equipments. Moreover, different masks need to be designed each time one needs to prepare a different patterned arrangement.

Concerning mask-less patterning techniques, one can distinguish between methods in which each patterned structure is generated individually in an in-series sequence (e.g., nanoindentation^8^, dip-pen nanolithography^9^, laser patterning^10^, focused ion beam irradiation^11^, etc.) and methods in which periodic patterns are generated spontaneously at once (e.g., spatio-temporal pattern formation in some electrodeposited binary systems^12^, self-assembly of block-copolymers^13^, anodization^14^, maze-like magnetic domains in films with perpendicular magnetic anisotropy^15^, self-assembled strain driven chemical solution derived nanostructures^16^, etc.). Compared to the in-series generation of individual patterned elements, the spontaneous formation of self-organized arrangements is much faster and offers the possibility of easily patterning large surface areas. Yet, precise control of self-organized patterning to obtain reproducible specific shapes or periodic arrays of structures with long-range order remains a challenging issue.

Spatio-temporal pattern formation is a topic not restricted to materials science. Actually, this phenomenon occurs frequently in nature, in open systems far from equilibrium, for example in the environment (atmosphere and oceans), in the skin of some animals (like in zebras or giraffes), in atrial fibrillation, in geophysical systems, etc. Spatio-temporal patterns are related to chaos and their study is applicable to a wide range of disciplines, such as crime analysis^17^, demography and epidemiology^18^, cell proliferation and tissue engineering^19^ and, of course, statistics and mathematics^20^.

Formation of spatio-temporal patterns in nature can stem from either a flux of energy or a flux of matter, e.g. originated from the flow of fluids or chemicals in a reactor. The latter is responsible for the emergence of spatio-temporal patterns during the electrodeposition of some binary systems, like Ag-Bi, Ag-Cd, Ag-In, Ag-Sb or Co-In^12^,^21^,^22^,^23^,^24^,^25^,^26^,^27^, leading to different areas having dissimilar local composition. Although the mechanism for spatio-temporal patterning during electrodeposition has not been fully elucidated yet, a variety of factors, such as the electrolyte hydrodynamic conditions, the overpotential, the enthalpy of mixing, kinetic variables and the appearance of Turing instabilities in reaction-diffusion systems seem to play a synergetic role on this phenomenon^12^,^28^. Archetypical morphologies observed in these electroplated materials include ‘labyrinths’, ‘waves’, ‘targets’, spirals, broken spirals and mixed patterns^12^. Unfortunately, since non-linearity and chaos are at the heart of spatio-temporal pattern formation, it is often difficult to control the shapes of the generated structures in electrodeposited continuous films, although some studies have reported certain variations of the pattern geometry depending on the film’s composition, thickness and electrodeposition conditions^12^.

Compared to the Ag-based alloys series, the electrodeposition of Co-In alloys has been far less explored. Pattern formation under both stagnant and high-speed electrodeposition from electrolytes containing sulphate metal salts and di-ammonium hydrogen citrate at pH around 3 has been well-established by Krastev *et al*.^27^. Typically, spatio-temporal structures form at sufficiently negative applied current densities (large Co contents). Since In and Co are immiscible in both solid and liquid states^29^, the formation of multi-phase coatings is indeed anticipated. Remarkably, we have recently demonstrated that topographic and compositional spatio-temporal patterning in electrodeposited Co-In films induces a concomitant periodic change of the surface physical properties^26^. That is, compositional patterning in this system is accompanied by magnetic and mechanical micropatterning over large surface areas. This is of significant practical importance since it can be used as a mask-less or template-free inexpensive technique to achieve magnetic/mechanical surface patterning.

In the particular case of magnetic properties, adjustable lateral compositional modulation can lead not only to a range of interesting fundamental magnetic properties (e.g., domain wall manipulation^30^,^31^,^32^,^33^), but also to appealing potential applications such as lateral giant magnetoresistance sensors^34^, magnetic remote motion control of nanoscale magnetic nanoparticles through microfluidic structures along specific stray field tracks^35^,^36^, magnetic biosensors^37^, magnetic encoders (e.g., for biomedical applications)^38^,^39^ or possible 3D-magnetic states when combined with multilayers^40^. However, any exploitation of spatio-temporal patterning for devices necessarily relies on the ability to control the shapes of the generated motifs.

In this work, we demonstrate that the generation of patterns by spatio-temporal self-organization can be controlled by electrodeposition in confined areas, i.e., disks commensurate with the typical size of the spatio-temporal features. The idea is to use the edges of these disks as a guide for the formation of spiral-like spatio-temporal patterns. The procedure is to some extent analogous to “directed self-assembly” in block copolymers in which aligned lamellas form inside pre-defined stripes^41^,^42^,^43^. When grown as micrometer-sized circular disks, Co-In alloys develop clear spiral patterns with Co-rich and In-rich areas. This compositional patterning leads to patterning of the physical (magnetic, electrical and mechanical) properties, which locally vary also following the spiral-like geometries.

## Results

### Morphological and compositional analysis

**Figure 1:**
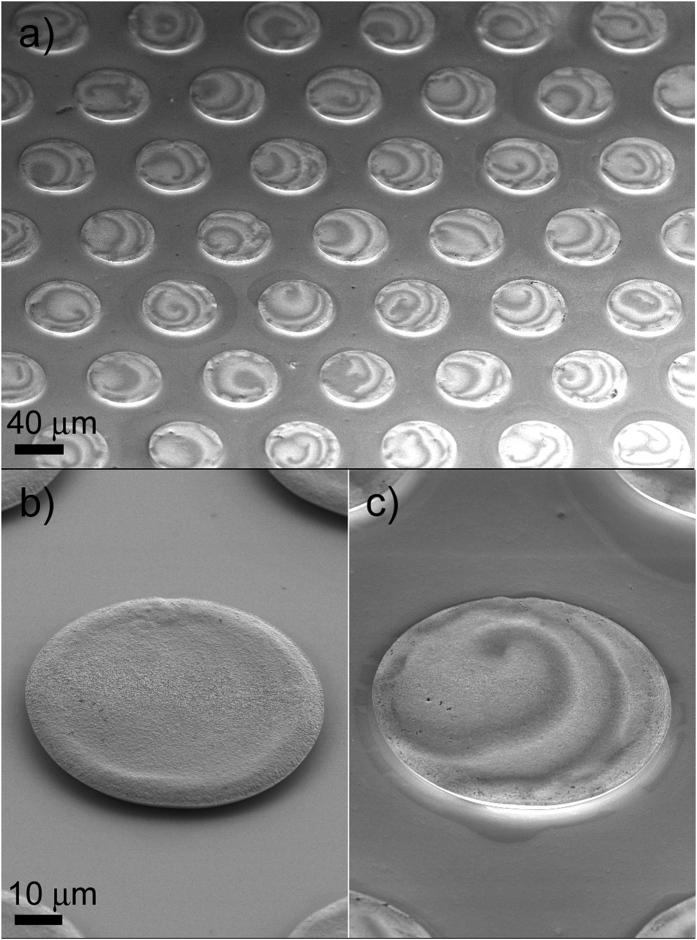
FE-SEM images of (**a**) an array of Co–In microdisks (taken with In-Lens detector), (**b**) detail of one microdisk taken with SE detector and (**c**) corresponding image obtained using the In-Lens detector.

Figure 1a shows a field emission scanning electron microscopy (FE-SEM) image of an array of electrodeposited Co-In microdisks using an In-Lens detector, which is sensitive to differences in the work function (i.e., electronic variations). A spiral-like pattern at the surface of each disk can be observed, although the exact shape of the spirals varies somewhat from one disk to the other. Figure 1b,c show the details of a single microdisk imaged using the secondary electrons (SE) and the In-Lens detectors, respectively. Remarkably, the spiral-like pattern is only revealed when using the In-Lens detector, not with the SE detector. Besides gathering information on morphology and surface topography of specimens, the In-Lens detector reveals differences in composition. On the contrary, topographic information predominates in any image recorded using the conventional SE detector. This indicates that the contrast in (a) and (c) is mainly due to the local changes in composition (i.e., Co/In stoichiometry) and distantly related to topographic variations (e.g., surface corrugation). This is in contrast to Co-In films, for which the surface spatio-temporal patterns were found to be linked to both topographical and compositional variations^26^. Since the films were several microns thick, probably the spatio-temporal topographic features had sufficient space to build up. Conversely, since microdisks are only 1 *μ*m thick (the maximum thickness of the disks cannot be larger than the thickness of the resist used in the optical lithography), the deposition time is likely not sufficiently long for the spirals to fully develop. As a result, the typical periodic topographical features observed in continuous thick Co-In films are not visible in the disks. Nonetheless, the SE and atomic force microscopy (AFM) images indicate that the microdisks are not completely flat. Actually, they are slightly thicker toward the edges (see Fig. 1b). This can be explained by a non-uniform distribution of the current density at the cylindrical cavities. Current density is typically higher at the edges, hence resulting in faster deposition rates^44^. However, it is clear that the strong contrast observed in Fig. 1a mainly stems from the differences in composition between the light and dark grey regions and it is not dominated by the surface topography.

**Figure 2:**
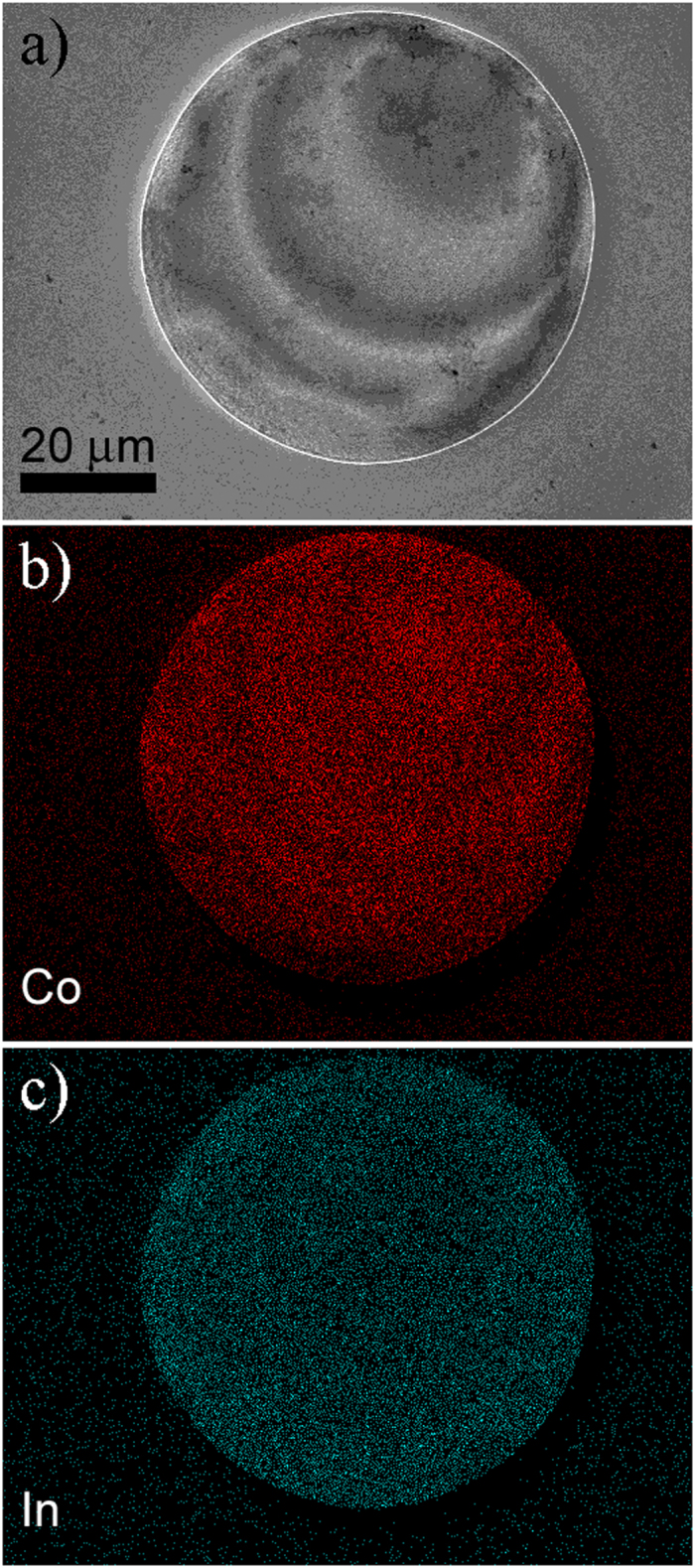
EDX mapping of a Co–In microdisk.

The exact elemental composition of the Co-In microstructures was determined by energy dispersive X-ray spectroscopy (EDX) in the SEM. The overall composition of the disks is 78 ± 1 at% Co and 22 ± 1 at% In, which is in accordance with previous works on Co-In films electrodeposited at j = −20 mA cm^−2^ for 1000 s^26^. The EDX mapping of the microdisks reveals that Co is not homogeneously distributed, but it follows the spiral-like pattern as previously observed by the SEM In-Lens detector imaging (Fig. 1). In particular, the darker areas in the In-Lens image are enriched in Co (Fig. 2b). On the other hand, the In distribution does not follow any obvious ‘complementary’ pattern to the Co one, but it rather seems that the In content increases toward the periphery of the disk (Fig. 2c). Thus, the compositional spiral pattern indicates a modulation of the Co content.

Remarkably, unlike the spatio-temporal patterns spontaneously formed over large areas, which typically show a mixture of spirals, targets and waves^12^,^26^, electrodeposition of Co-In in confined areas lead to a preferential pattern shape, i.e., spirals in the case of disks. This spontaneous self-assembled chemical patterning should be related to the lateral constraint imposed by the cylindrical micron sized cavities. However, to optimize the pattern control the size of the confined electrodeposition space needs to be commensurate with the size of typical features of the spatio-temporal patterning (which depend on the type of alloy and its composition). For example, in Co_78_In_22_ films the size of the typical topographic-compositional motifs is of a few microns^26^. Thus, the size of the constraint growth area should tentatively be of tens of microns. The guided process for pattern formation with controlled geometry could be thought to be conceptually analogous to the so-called “directed self-assembly” in block copolymers to form aligned lamellas inside pre-defined stripes^41^,^42^,^43^.

The modulated Co/In composition should result in distinct local physico-chemical properties. Thus, the electrical, magnetic and nanomechanical behavior, as well as the surface potential of the microdisks, were further explored.

### Electrical characterization

**Figure 3:**
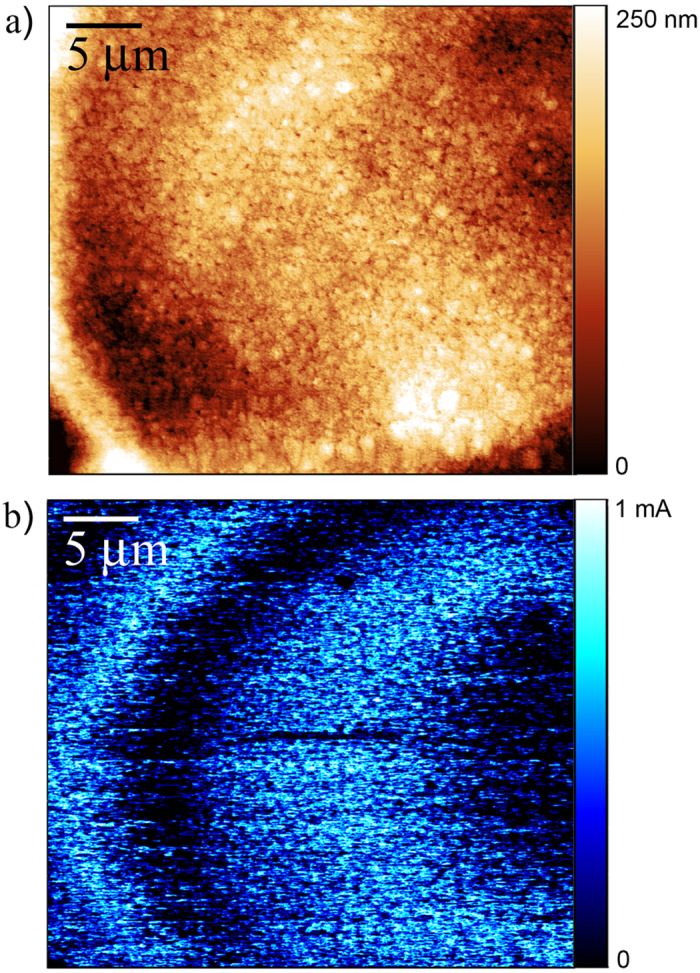
(**a**) AFM topographical and (**b**) current map image taken with the C-AFM under a constant applied load of 50 nN on 35 × 31 *μ*m^2^ scanned area of a Co-In microdisk.

Conductive atomic force microscopy (C-AFM) measurements using a bias voltage of 500 mN confirmed the presence of two different conductive domains, as shown in Fig. 3, being the current ratio between the bright (Co-rich) and dark (In-rich) regions about 100–10. These domains are not directly correlated to the surface topology (compare Fig. 3a,b) but to the aforementioned compositional variations. The occurrence of these domains with such large variation in conductivity cannot be simply explained based on the dissimilar electrical resistivity values for bulk Co (6.2 *μ*Ω cm at 20 °C)^45^ and bulk In (8.8 *μ*Ω cm at 20 °C)^46^. In fact, X-ray diffraction analyses of continuous films indicated the co-existence of several phases: tetragonal In, face-centered cubic Co, hexagonal close-packed Co, tetragonal CoIn_3_ and even an amorphous fraction in the deposits^26^. It has been argued that the formation of the intermetallic CoIn_3_ phase is the key toward the formation of spatio-temporal features in this type of deposits^47^. Although no data on the electrical conductivity/resistance is available for CoIn_3_ phase, it is known that intermetallic phases possess much higher electrical resistance than their pure constituents^48^. Therefore, it can be argued that the accumulation of CoIn_3_ at certain areas could contribute to the presence of the markedly low current domains (i.e., dark blue regions in Fig. 2b). Note that while Fig. 3a shows the topography of the disk at the surface, the current map image (Fig. 3b) is obtained from the contribution of the overall thickness of the disk to the electrical resistivity. Hence, a one-to-one coincidence between the two images should not necessarily be anticipated.

### Magnetic behavior

**Figure 4:**
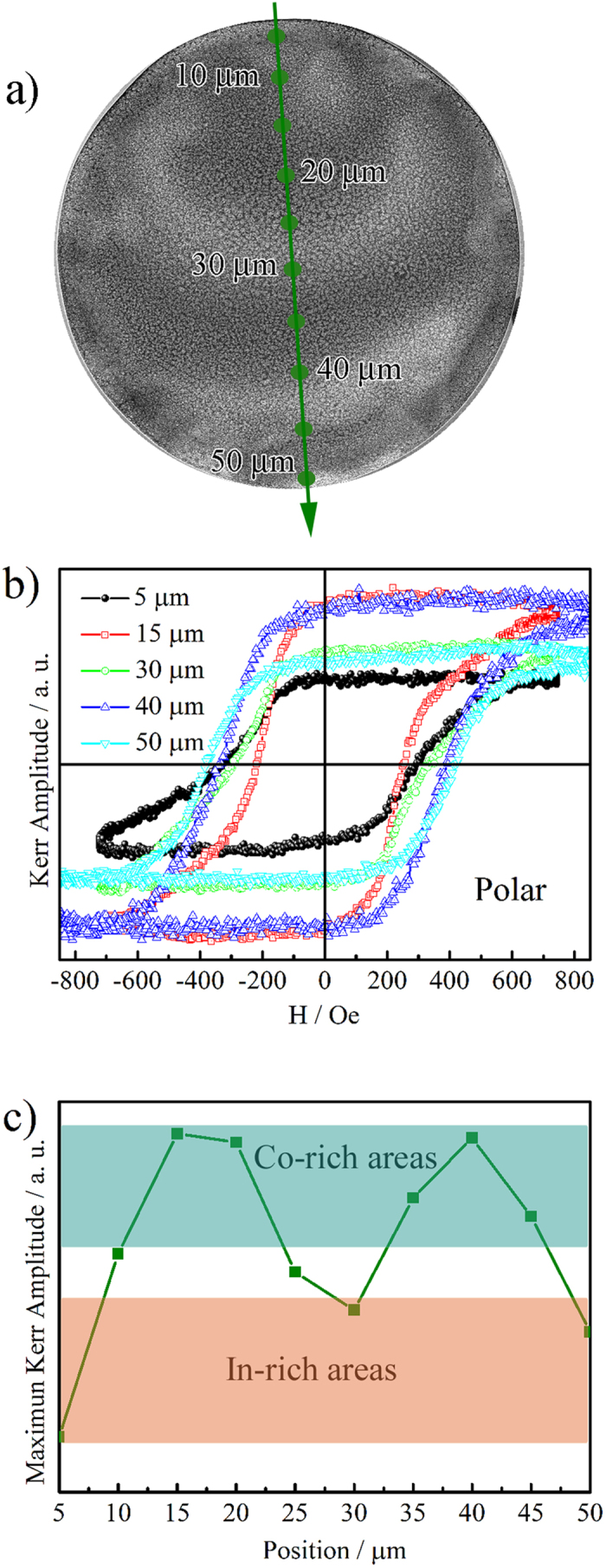
(**a**) In-Lens SEM image of a Co-In microdisk showing the positions onto which the MOKE laser was focused. (**b**) Polar (out-of-plane) room-temperature MOKE magnetic hysteresis loops for different positions across the diameter of the disk. (**c**) Dependence of the maximum Kerr amplitude as a function of position.

The local magnetic properties of individual Co-In microdisks were measured by magneto-optic Kerr effect (MOKE) in polar (out-of-plane) configuration at room temperature. The surface of the disk was probed every 5 *μ*m along its diameter with a laser spot of 3 *μ*m. Hysteresis loops were recorded at each location in order to reveal possible variations in the saturation magnetization (M_*S*_) and/or the coercivity (H_*C*_) (Fig. 4). Results show that the Co-rich regions exhibit a higher Kerr effect amplitude, which can be considered proportional to M_*S*_. Actually, M_*S*_ periodically varies as the Co-rich and the Co-depleted regions are being scanned by the laser, hence indicating an obvious ‘magnetic micro-patterning’. In addition, H_*C*_ shows values ranging between 200 to 400 Oe. The correlation between H_*C*_ and the composition is less obvious than that of M_*S*_, probably because H_*C*_ depends not only on the Co content but also on other microstructural parameters (crystallographic phases, grain sizes, microstrains, etc.) which play a role on the propagation of domain walls and on the overall magnetization reversal process. Nonetheless, the H_*C*_ values of the microdisks are considerably higher than those of films featuring analogous composition (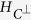 = 136 Oe)^26^. This increase in H_*C*_ compared to the unpatterned film is possibly due to domain wall hindrances caused by the constrained lateral size of the dots and their reduced thickness^7^,^49^,^50^.

**Figure 5:**
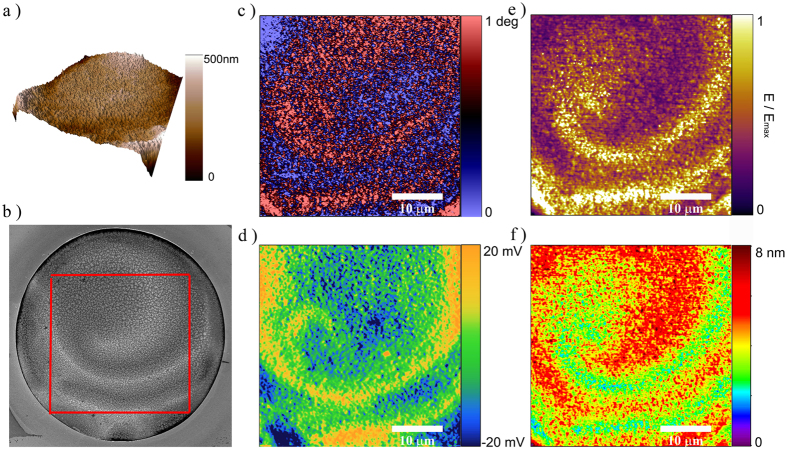
(**a**) AFM topography and (**b**) In-Lens SEM image of the Co-In microdisk. (**c**) MFM image at remanence after saturation at 1 T in plane. The image was taken in double pass mode, by scanning at a lift height of 30 nm with the same amplitude of oscillation as that used as setpoint for topography. Mapping of (**d**) work function acquired by KPFM using a tip voltage of 1 V, (**e**) normalized Young’s modulus and, (**f**) penetration depth (in nm) measured in AM-FM viscoelastic mapping mode.

The occurrence of a ‘magnetic micro-patterning’ was further studied by magnetic force microscopy (MFM) at remanence after having saturated the sample in the in-plane direction applying a magnetic field of 10 kOe. Figure 5 shows a comparison of the topography, SEM compositional contrast and different AFM modes corresponding to the same Co-In disk. The MFM image in Fig. 5c shows a clear magnetic contrast, stemming from the strength of the stray fields over the different scanned areas, which should be proportional to the magnetization. The results are in accordance with MOKE measurements. Namely, the spiral-like pattern is clearly disclosed, in which regions of higher M_*S*_ are alternated with regions of lower M_*S*_ in a helicoidal fashion.

**Figure 6:**
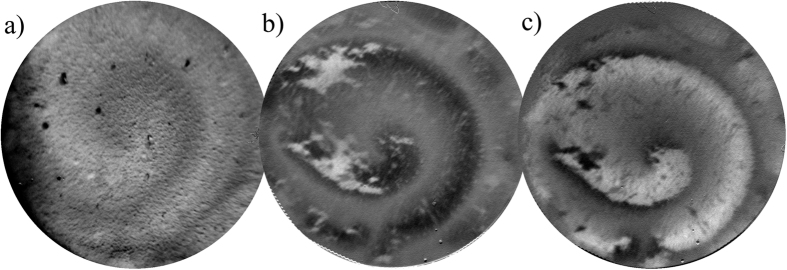
(**a**) XAS-PEEM (elemental map) and (**b,c**) XMCD-PEEM images at the Co L-edge. (**b,c**) are measured in remanence after applying a saturating field (650 Oe) in the (**b**) positive (+M_*R*_) and (**c**) negative (−M_*R*_) in-plane direction.

Additional evidence for the magnetic micro-patterning can be obtained from X-ray photoemission electron microscopy (PEEM), which is both element-specific and sensitive to the magnetization direction^51^. The X-ray absorption spectroscopy (XAS)-PEEM image at the Co L_3_ edge (Fig. 6a) shows the expected compositional pattern, in agreement with the SEM In-Lens and EDX characterization.

The X-ray magnetic circular dichroism (XMCD)-PEEM image at the same edge, also displays a spiral-like pattern. At remanence (after having applied a in-plane magnetic field of 650 Oe), the Co-rich areas show a majority of domains (either black or white) pointing along the previous saturation field, which has opposite sign for Fig. 6b,c. In both images there are small reverse domains (small dots or stripes) within the majority, which are most likely formed during the relaxation from saturation to remanence. Some of these reverse domains are exactly at the same position in both images indicating that they are most likely pinned to structurally or compositionally different seed areas. Furthermore, a few domains are not reversed between the two images, suggesting that there are some magnetically hard regions, which cannot be switched by the applied in-plane field (±650 Oe). The PEEM observations corroborate the magnetic micro-patterning observed by MOKE and MFM. In addition, the correlation between the composition and magnetic properties becomes also evident when comparing the XAS with the XMCD PEEM images. Remarkably, the various magnetic techniques used in this study have different probing depths and lateral resolutions: PEEM (5 nm/100 nm), MOKE (20–30 nm/3 *μ*m) and MFM (stray fields/50–100 nm). Hence, they provide complementary information and evidence that a net magnetic patterning probably extends from the surface down to several hundred nm.

### Local compositional analysis by work-function and nanomechanical characterization

The work-function of a Co-In microdisk was mapped by Kelvin probe force microscopy (KPFM) and the images are shown in Fig. 5, together with AFM and MFM images recorded on exactly the same area (and the same dot). Since, Co and In have different work-functions (W(Co) = 5 eV and W(In) = 4.09 eV), changes in the KPFM images can be anticipated. Thus, the image in Fig. 5d corroborates the already assessed local modulation of the composition (Figs 1c and 2). Indeed, as for the other properties, the work function mapping is able to perfectly reconstruct the spiral-like pattern (Fig. 5d), confirming the EDX and In-Lens imaging results.

Finally, the mapping of the Young’s modulus, E, obtained from amplitude modulation - frequency modulation (AM-FM) viscoelastic mapping in the same area (Fig. 5e) shows a clear contrast with spiral-like symmetry, where the Co-rich areas correspond to the lighter color (yellowish), which indicate higher Young’s modulus. Similar trends were observed for the penetration depth AFM map (Fig. 5f). The results indicate that the Co-rich areas are mechanically harder (larger E and smaller penetration) than the In-rich ones, in agreement to what has been found in thin films by nanoindentation^26^. Owing to the different mechanical properties of hcp/fcc-Co (with average Young’s modulus around 210 GPa in non-porous bulk form ref. 52), and In (Young’s modulus around 12.5 GPa^53^), the chemical patterning on the surface of the microdisks can explain the variations in the elastic modulus (Fig. 5e) and indentation depth (Fig. 5f), even if the exact distribution of the crystallographic phases within the patterns is not clear at present. Namely, even if the nanomechanical properties in In-rich regions could be influenced by the presence of intermetallic CoIn_3_ (presumably with a relatively high Young’s modulus), the amount of pure In phase accompanying CoIn_3_ is likely higher in these regions^26^. Moreover, similar to the magnetic properties, there is an evident one-to-one correlation between the composition (work function) and mechanical properties.

## Discussion

In conclusion, we have demonstrated that the morphology of spatio-temporal patterns in electrodeposited Co-In alloys can be adjusted by confining their growth. When electrodepositing in arrays of circular cavities of 50 *μ*m in diameter, Co-In microdisks with spiral-like compositional variations are obtained. The local changes in composition lead to a periodic modulation of the physical (electric, magnetic and mechanical) properties, with a univocal correlation between composition and the resulting properties. The Co-rich areas are mechanically harder, more electrically conducting and exhibit higher saturation magnetization than the In-rich ones. The ability to control the pattern formation in spatio-temporal systems by confined growth may not only pave the way to possible new applications of electrodeposited alloys (e.g., encoders or magnonic crystals), but it may also open new research avenues in the many disciplines in which spatio-temporal patterns play an important role (e.g., biology, medicine or even social behavior).

## Methods

Electrodeposition of Co-In was performed onto pre-lithographed Si (111) substrates with evaporated Ti (100 nm)/Au (400 nm) adhesion/seed layers. Arrays of cylindrical holes of 50 *μ*m in diameter and 1 *μ*m in height were first patterned by photolithography using AZ-9260 photoresist and AZ-400K developer. The patterned area was used as a mask for the subsequent electrodeposition of the Co-In microdisks. A one-compartment thermostatized three-electrode cell connected to a PGSTAT302N Autolab potentiostat/galvanostat (Ecochemie) was employed. Deposition was conducted potentiostatically at −0.98 V in an electrolyte containing 0.05 M *InCl*_3_ + 0.25 M *CoCl*_2_ + 0.05 M *C*_6_*H*_5_*Na*_3_*O*_7_•2*H*_2_*O* + 0.1 M *KCl*. Bath temperature was kept at 25 °C. The electrolyte was prepared from analytical grade reagents and Milipore Milli-Q water (18 MΩcm). A representative current-time curve is shown in the S.I. The morphology and the composition of the Co-In microdisks were studied on a Zeiss Merlin FE-SEM equipped with an EDX detector. Room-temperature magnetic hysteresis loops were recorded using a MOKE setup (Durham Magneto Optics) with a maximum field of 800 Oe, applied out-of-plane (polar configuration). Note that to measure the local magnetic properties, the MOKE laser spot was focused down to 3 *μ*m onto different regions of the Co-In microdisks. C-AFM measurements were performed with a commercial AFM (Agilent 5500LS) operating in contact mode with a commercial tip from AppNano (DD-SICONA) and with k = 0.2 N/m. A bias voltage of V_*bias*_ = 500 mV was applied to the sample while the tip was grounded. An Asylum AFM (MFP3D) operated in two different modes was used for the nanomechanical and magnetic characterization and surface potential function mapping. AM-FM viscoelastic mapping was used for the nanomechanical characterization. In this mode the cantilever is driven simultaneously at two excitation signals that are combined to excite the fundamental and second cantilever resonances. While the topography is recorded in normal AM mode at the fundamental resonance, the second resonance frequency is driven in FM, and the measured changes in frequency and dissipation of this resonance are correlated to loss tangent, sample stiffness and elasticity, and viscous behavior or dissipation. To map the different surface potential function, KPFM mode was used. Finally, to visualize the magnetic domains, a MFM study was performed in a dual-scan mode using a magnetized tip (Nanosensors MFMR tip with k = 2 N/m and a CoCr coating of 35 nm). Finally, compositional (XAS) and magnetic contrast imaging (XMCD) using element-specific polarized XPEEM experiments were carried out in beamline BL24 – CIRCE at the ALBA Synchrotron^54^. To construct the chemical or magnetic element-specific figures two images taken with opposite photon helicity are either added or subtracted pixel-by-pixel, respectively.

## Additional Information

**How to cite this article**: Golvano-Escobal, I. *et al*. Spontaneous formation of spiral-like patterns with distinct periodic physical properties by confined electrodeposition of Co-In disks. *Sci. Rep.* **6**, 30398; doi: 10.1038/srep30398 (2016).

## Supplementary Material

Supplementary Information
