# Supplementary material for: Spontaneous formation of spiral-like patterns with distinct periodic physical properties by confined electrodeposition of Co-In disks
Source: Sci Rep. 2016 Jul 27;6:30398. doi: 10.1038/srep30398 (PMC4961954; doi:10.1038/srep30398)
Supplement: Supplementary Information [file srep30398-s1.pdf]

Spontaneous formation of spiral-like patterns with distinct periodic physical properties by confined electrodeposition of Co-In disks

Irati Golvano-Escobal[1], Juan Carlos Gonzalez-Rosillo[2], Neus Domingo[3], Xavi Illa[4,5], José Francisco López-Barbera[1,3], Jordina Fornell[1], Pau Solsona[1], Lucia Aballe[6], Michael Foerster[6], Santiago Suriñach[1], Maria Dolors Baró[1], Teresa Puig[2], Salvador Pané[7], Josep Nogués[3, 8], Eva Pellicer[1,], Jordi Sort[1, 8,*]*

[1]Departament de Física, Universitat Autònoma de Barcelona, E-08193 Bellaterra, Spain

[2]Institut de Ciència de Materials de Barcelona (ICMAB-CSIC), Campus UAB, E-08193 Bellaterra, Spain

[3]Catalan Institute of Nanoscience and Nanotechnology (ICN2), CSIC and The Barcelona Institute of Science and Technology, Campus UAB, Bellaterra, E-08193 Barcelona, Spain

[4]Institute of Microelectronics of Barcelona (IMB-CNM), Campus UAB, E-08193 Bellaterra, Spain

[5]Biomedical Research Networking Center in Bioengineering, Biomaterials and Nanomedicine (CIBER-BBN), E-08193, Bellaterra, Spain

[6]Alba Synchrotron Light Facility, CELLS, E-08280 Bellaterra, Spain

[7]Institute of Robotics and Intelligent Systems (IRIS), ETH Zürich, CH-8092 Zürich, Switzerland

[8]Institució Catalana de Recerca i Estudis Avançats (ICREA), E-08010 Barcelona, Spain

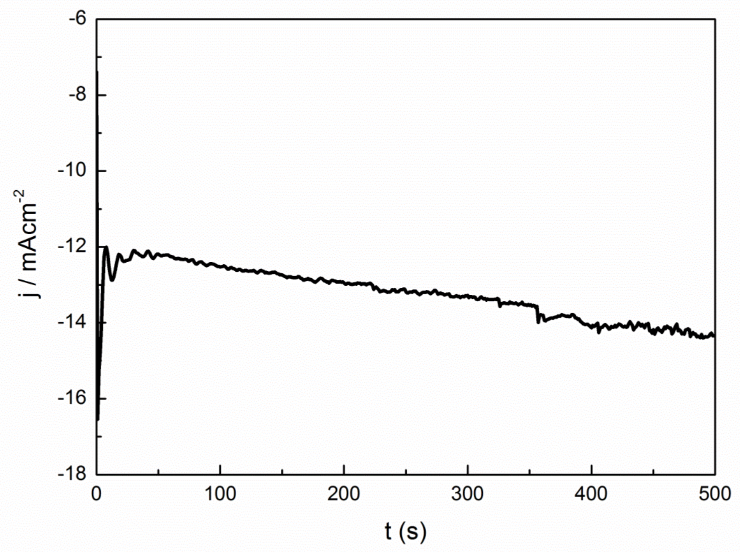

Figure S1. Potentiostatic curve for Co-In alloy micro-disks deposition onto pre-lithographed Au/Ti/Si substrates at –0.98 V .
